# Highly Conserved Influenza A Nucleoprotein as a Target for Broad-Spectrum Intervention: Characterization of a Monoclonal Antibody with Pan-Influenza Reactivity

**DOI:** 10.3390/vetsci13010045

**Published:** 2026-01-03

**Authors:** Jingrui Liu, Wenming Gao, Kunkun Zhao, Zongmei Huang, Lin Liu, Jingjing Chang, Xiaoyang Cao, Wenwen Zhou, Xiaojie Zhou, Yuman Liu, Xinsheng Li, Yapeng Song

**Affiliations:** College of Veterinary Medicine, Henan Agricultural University, Zhengzhou 450046, China; l2639402115@outlook.com (J.L.); 15803826941@126.com (W.G.); zkjyzkk@163.com (K.Z.); ndkjchung@163.com (Z.H.); 15638870010@163.com (L.L.); c2640460041@163.com (J.C.); c13623981383@outlook.com (X.C.); zwenwen1805@163.com (W.Z.); 18738701513@163.com (X.Z.); 13673370901@163.com (Y.L.)

**Keywords:** influenza A virus, NP protein, mAb, conserved epitope, antiviral target

## Abstract

Influenza A viruses remain a persistent global health challenge due to their rapid antigenic evolution, zoonotic potential, and pandemic threat. Universal countermeasures targeting conserved viral components are urgently needed to enhance diagnostic, surveillance, and therapeutic capabilities. Here, we report the generation and characterization of a high-affinity monoclonal antibody (2D8 mAb) against the nucleoprotein (NP) of the H9N2 avian influenza virus, a subtype with increasing relevance to human infections. Importantly, 2D8 mAb exhibited robust cross-reactivity with a broad spectrum of influenza A viruses, including H1, H3, H5, H7, and H9 subtypes, while showing no cross-reactivity with unrelated viral pathogens. Epitope mapping identified its binding target as a highly conserved NP motif ^38^RFYIQMCTEL^47^, which is invariant across all major human influenza A lineages. Isotyping revealed 2D8 mAb to be of the IgG2b/κ subclass, with an exceptionally high titer (1:20,480,000) as determined by ELISA. Given the essential role of NP in viral replication and host adaptation, this antibody offers a powerful platform for next-generation diagnostic assays capable of detecting a wide range of human and zoonotic influenza A viruses using a single reagent. Moreover, it holds potential for guiding the design of universal antiviral strategies targeting structurally constrained regions of the influenza virus. Our findings provide a valuable resource for advancing pan-influenza A interventions, with direct implications for improving pandemic preparedness and strengthening global influenza surveillance in both clinical and public health settings.

## Simple Summary

The influenza A virus poses a persistent global threat to human health and the poultry industry, as it evolves rapidly and can cross species to spread. To enhance our ability to track these viruses, scientists are seeking “universal” tools that can identify multiple different influenza virus strains at once. In this study, we developed a special monoclonal antibody named 2D8, which targets the NP protein of the influenza virus. This protein is a part of the virus that remains almost unchanged across different influenza subtypes. Our tests showed that this antibody can identify multiple influenza subtypes, including those found in birds, pigs, and humans, such as H1, H3, H5, H7, and H9. We also identified the precise antigenic epitope where this antibody binds to the virus and confirmed that this site is almost identical in 35 different virus samples from around the world. These findings are of great significance to society, as they provide a blueprint for creating faster and more reliable diagnostic tests and help in developing “universal” vaccines that can prevent multiple influenza types with just one injection.

## 1. Introduction

Influenza A viruses, a major global health threat, are single-stranded, negative-sense RNA viruses belonging to the Orthomyxoviridae family [1]. These viruses are classified into subtypes based on the antigenic properties of their surface glycoproteins, hemagglutinin (HA), and neuraminidase (NA), with 16 HA and 9 NA subtypes identified across a range of hosts, including humans, birds, and mammals [2]. Human influenza A viruses, notably subtypes H1N1, H3N2, and seasonal variants, contribute to significant morbidity and mortality worldwide, exacerbating the global burden of respiratory diseases. In particular, the ongoing evolution of influenza A viruses, coupled with their ability to cross species barriers and reassort with avian strains, poses continuous threats to public health, as seen in recent years with novel influenza variants [3,4].

One of the most concerning subtypes of avian influenza virus (AIV), H9N2, has demonstrated zoonotic potential and has been associated with human infections in various regions [5,6,7,8,9,10,11]. Over time, H9N2 has undergone significant genetic diversification and recombination with other influenza subtypes, enhancing its ability to infect both poultry and humans [12,13,14,15,16,17,18,19,20,21]. Although vaccines targeting H9N2 have been used in the poultry industry, their effectiveness has waned over time due to viral mutations and antigenic drift, underscoring the urgent need for broad-spectrum diagnostic and therapeutic solutions that can provide protection against a wide range of influenza A subtypes.

The nucleoprotein (NP) of influenza A viruses is a highly conserved, internal protein essential for viral replication. It is one of the most abundant viral proteins and plays a critical role in the assembly and functioning of the viral ribonucleoprotein (vRNP) complex, which is required for the replication, transcription, and packaging of the viral genome [22,23,24,25,26]. Due to its fundamental role in the virus life cycle and its relative conservation across influenza A subtypes, the NP protein has emerged as a promising target for the development of universal diagnostics and broad-spectrum vaccines. In fact, diagnostic assays based on monoclonal antibodies (mAbs) targeting the NP protein have demonstrated the ability to recognize diverse influenza A subtypes, facilitating early detection and surveillance of both seasonal and emerging strains [27].

Studies have shown that anti-NP antibodies cross-react with a wide range of influenza A strains, including those from avian, swine, and human origins [28]. The conserved nature of the NP protein across influenza A subtypes allows for the development of diagnostic tools that can detect multiple viral strains with a single reagent, regardless of their HA or NA antigenic variation. Furthermore, NP-based vaccines have demonstrated broad protective potential against various influenza A strains, making them an attractive target for universal influenza vaccines [29,30,31,32,33].

In this study, we focused on the development of a mAb targeting the NP protein of H9N2 AIV, with the aim of expanding its applicability to human influenza viruses. By immunizing mice with the H9N2 strain, monoclonal antibodies were prepared. The broad spectrum and specificity of the monoclonal antibody were verified through various methods. The conservation of the recognized B-cell epitopes was determined through epitope mapping and homology analysis, offering insights into the potential for universal diagnostics. This research provides foundational knowledge for the development of mAb-based diagnostic assays that can accurately detect influenza A infections across species, aiding in both surveillance and early detection of emerging strains.

## 2. Materials and Methods

### 2.1. Cell Lines, Viruses, and Animals

Madin-Darby canine kidney (MDCK) cells were cultivated in Dulbecco’s modified Eagle’s medium (BioChannel, Nanjing, China), supplemented with 10% fetal bovine serum (KEL Biotech, Shanghai, China). Mouse myeloma cells (SP2/0) were cultured in hybridoma cell serum-free medium (BasalMedia, Shanghai, China) at 37 °C with 5% CO_2_. The MDCK cell line (ATCC Cat # CCL-34) and SP2/0 cell line (ATCC Cat # CRL-1581) were obtained from the American Type Culture Collection (ATCC, Manassas, VA, USA). The following strains of influenza virus have been identified: avian influenza virus H9N2 subtype HN22 strain (A/chicken/China/HN22/2022) (GenBank accession number: PP030808-PP030815); avian influenza virus H9N2 subtype SQ2023 strain (A/chicken/Shangqiu/SQ2023/2023) (GenBank accession number: PP940140-PP940147); avian influenza virus H3N3 subtype SQ2049 strain (A/chicken/Henan Shangqiu/SQ2049/2023) (GenBank accession number: PP758466-PP758473); swine influenza virus H1N1 subtype ZC90 strain (A/swine/Zhucheng/90/2014) (GenBank accession number: KX264367-KX264374); the infectious bronchitis virus C2023-03 strain (CK/CH/HN/YZ202303) (GenBank accession number: PP504785); and the fowl aviadenovirus WZ strain (FAdV-WZ) (GenBank accession number: MZ508442.1). In the present study, three strains of viruses were preserved in the laboratory, namely, the fowl aviadenovirus HN1472 strain (FAdV-HN1472) (GenBank accession number: OR975470.1), the duck adenovirus GD2588 strain (DAdV-GD2588) (GenBank accession number: PV988401), and the chicken anemia virus HN2021-1412 strain (CIAV-1412) (GenBank accession number: MZ369153). The hemagglutination inhibition test antigens of the following influenza virus subtypes were procured from Harbin Guosheng Biotechnology Co., Ltd. (Harbin, China): H5 (Re-13 strain and Re-14 strain) and H7 (Re-4 strain). The hemagglutination inhibition test antigens of the following virus were procured from Beijing Zhonghai Biotech Co., Ltd. (Beijing, China): the egg drop syndrome virus 911 strain (EDSV-911) and the Newcastle disease virus La Sota strain (NDV-La Sota). Six 6–8-week-old BALB/c mice were obtained from Changchun Changsheng Biotechnology Co., Ltd. (Changchun, China). Among them, three were used as the immunization group and the other three as the control group. Specific pathogen-free (SPF) chicken embryos aged 9–11 days were purchased from Beijing Boehringer Ingelheim Vital Biotechnology Co., Ltd. (Beijing, China). All immunization procedures adhered to the institutional animal care and use guidelines (Animal Welfare and Ethics Approval No. HNND2024052225).

### 2.2. The Isolation of the Virus Strain

The H9N2-HN22 strain was isolated from diseased tissues of a chicken farm in Henan Province and purified by SPF chicken embryos. The purified virus was then serially diluted 10-fold with phosphate-buffered saline (PBS) and inoculated into 9–11-day-old SPF chicken embryos at a dose of 0.2 mL per embryo. The embryonic chicken eggs were cultured in an incubator set at 37 °C, and observations were conducted up to 120 h post-inoculation. The hemagglutination (HA) titers of the allantoic fluid were determined for each chicken embryo, and the 50% egg infectious dose (EID_50_) and 50% lethal dose (ELD_50_) of the virus were calculated using the Reed–Muench method. The virus was serially diluted 10-fold and inoculated into MDCK cells. Following a 72 h incubation period at 37 °C within an incubator, the cell culture medium was harvested for the purpose of determining the HA titers. The 50% tissue culture infectious dose (TCID_50_) was subsequently calculated in accordance with the Reed–Muench method.

### 2.3. Genome Sequencing and Analysis

The viral RNA was extracted according to the instructions for the FastPure Viral DNA/RNA Mini Kit (Vazyme, Nanjing, China) reagent kit and then reverse-transcribed into cDNA using the HiScript II Q Select RT SuperMix for qPCR (+gDNA wiper) (Vazyme, Nanjing, China) reagent kit. Whole-genome amplification was performed using the primers designed by Hoffmann [34], and the amplification results were sent to Sangon Biotech (Shanghai) Co., Ltd. (Shanghai, China) for Sanger sequencing. Phylogenetic analysis was then carried out using the MEGA 7.0 software.

### 2.4. Mouse Immunization

The viral precipitates were resuspended in PBS and then incubated with 0.1% formaldehyde at 37 °C and oscillated at 180 rpm for 72 h to inactivate the virus. The inactivated virus was then filtered and sterilized using a 0.22 µm filter before being stored at −80 °C.

Female BALB/c mice were injected subcutaneously with the inactivated H9N2-HN22 strain mixed with Freund’s complete adjuvant (Sigma-Aldrich, St. Louis, MO, USA) at a volume ratio of 1:1. The mice were injected with 300 μL per mouse at the first immunization and then boosted with the same dose at two-week intervals. Serum was collected, and the serum titer was assessed using an ELISA. The mouse with the highest antibody level was further injected with a total of 300 μL of antigen intraperitoneally, three days prior to cell fusion, as part of the final immunization. Subsequently, splenocytes were aseptically extracted and fused with SP2/0 cells using 50% (*w*/*v*) PEG 1500 (Roche, Basel, Switzerland). The fused cells were cultured in 96-well plates in hybridoma cell serum-free medium with HAT for 10 days. Positive hybridoma cell lines identified by ELISA were subcloned three times by limited dilution. The mAb subtypes were identified using a mouse monoclonal antibody-isotyping ELISA kit (Proteintech, Wuhan, China), according to the manufacturer’s instructions. The specificity and reactivity of the antibodies were detected using Western blot and immunofluorescence assay.

### 2.5. Indirect Enzyme-Linked Immunosorbent Assay (iELISA)

An indirect ELISA was used to measure antibody levels in the supernatant of hybridoma cells. Briefly, ELISA plates were coated with inactivated H9N2-HN22 virus and incubated at 37 °C for 2 h and at 4 °C overnight. After three washes with PBST buffer, the plates were blocked with 5% skimmed milk at 37 °C for 2 h.

Following three more washes with PBST, the hybridoma cell culture supernatants were added to the plates and incubated at 37 °C for 1 h. After three more washes with PBST, the plates were incubated with HRP-conjugated goat anti-mouse IgG (H + L) (Proteintech, Wuhan, China) at 37 °C for 1 h. Following another three washes, TMB substrate (Solarbio, China) was added to the wells, and the reaction was carried out for 15 min before being terminated with 2 M H_2_SO_4_. The absorbance value was then detected at OD_450_ nm using an enzyme meter. In the course of the experiment, the reliability of the experiment was improved by setting up three replicate groups.

### 2.6. Subtype Identification

Mouse monoclonal antibody isotyping was performed using the mouse monoclonal antibody-isotyping ELISA kit (Proteintech, Wuhan, China), according to the manufacturer’s protocol, to determine the subtypes of selected mAb.

### 2.7. Western Blot

The collected samples were mixed with 5× protein loading buffer and boiled for 10 min. The proteins in the samples were separated by SDS-PAGE electrophoresis at 10% and transferred to nitrocellulose membranes (NC membranes) (Cytiva, Dassel, Germany), which were blocked with 5% skimmed milk at room temperature (RT) for 2 h. The NC membranes were then incubated with monoclonal or tagged antibodies screened with positive sera for 2 h at RT or overnight at 4 °C. After three washes with TBST, the membranes were incubated with HRP-conjugated goat anti-mouse IgG (H + L) as the secondary antibody at RT for 2 h at a dilution of 1:5000. Following three additional washes with TBST, the target proteins were detected using an Ultrasensitive ECL Detection Kit (Proteintech, Wuhan, China) and a Western blot imaging system (Cytiva, Tokyo, Japan).

### 2.8. Indirect Immunofluorescence Assay (IFA)

To identify the specificity of the anti-H9N2 AIV mAb, an immunofluorescence assay (IFA) was performed on MDCK cells infected with H9N2 AIV and H3N3 AIV. After 48 h, the cells were fixed with 4% paraformaldehyde for 30 min and permeabilized with a solution of 0.5% Triton X-100 for 15 min. Following an overnight incubation with 5% skim milk at 4 °C, the antibodies were added and incubated at RT for 1 h. After three washes with PBS, the cells were incubated with Dy-Light 488-conjugated goat anti-mouse IgG (H + L) (Abbikne, Wuhan, China) at a dilution of 1:5000 for 1 h. After staining with a 4′,6-diamidino-2-phenylindole (DAPI) solution (Solarbio, Beijing, China) for 15 min, the cells were imaged using a fluorescence microscope (Thermo Fisher Scientific, Waltham, MA, USA).

### 2.9. Dot Blot

The NP protein peptides were applied to the NC membrane, with PBS acting as the negative control and the isolate acting as the positive control. The samples were covered with 5% skimmed milk at RT for 1 h. After three washes with TBST, the NC membranes were then incubated with the antibodies screened with positive sera for 1 h at RT. After three further TBST washes, the membranes were treated with a 1:5000 dilution of HRP-conjugated goat anti-mouse IgG (H + L) as the secondary antibody for 1 h. The target proteins were detected using an Ultrasensitive ECL Detection Kit and a Western blot imaging system.

### 2.10. Truncation and Expression of NP Genes

The H9N2-HN22 NP protein was cloned into the pET-28a (+) vector and then transformed into BL21 (DE3) for expression. To find the B-cell epitope of this NP mAb, we used truncated primers based on the NP gene sequence of the isolate. To achieve a consistent size of the truncated fragments, two distinct expression vectors, pET-28a (+) and pET-32a (+), were employed. The truncated NP protein was divided into three pieces: NP-1, NP-2, and NP-3. These fragments were copied into the pET-28a (+) vector and then transformed into BL21 (DE3) for expression. The Western blot analysis was performed using the mAb and the His-tagged antibody, as explained earlier. To further locate the antigenic epitope, NP-4, NP-5, and NP-6 were cloned into the pET-32a (+) for expression, followed by Western blot analysis. In order to work out the antigenic epitopes, different peptides were made bit and bit, and the antigenic sites of the mAb were found using Dot Blot and ELISA. The primers used for the above-mentioned NP-truncating genes are shown in Table 1.

### 2.11. Further Localization of the Amino Acid Sequence of the Linear B-Cell Epitope

Some positive fragments were found after the first truncation. We then made more of these fragments through further truncation. The peptides made using this method were then identified by ELISA and Dot Blot. The peptides were synthesized by GL Biochem (Shanghai) Ltd. (Shanghai, China).

### 2.12. H9N2-HN22 NP Protein Structure Prediction and Conservation Analysis of Antigenic Epitopes

Online websites (https://novopro.cn/tools/secondary-structure-prediction.html (accessed on 18 May 2025)) were used to predict the secondary structure of the H9N2-HN22 NP protein and analyze the characteristics of the selected epitopes. The three-dimensional structure of the H9N2-HN22 NP protein was predicted using the SWISS-MODLE websites (https://swissmodel.expasy.org/ (accessed on 18 July 2025)), and the selected antigenic epitopes were displayed on the 3D structure of the NP protein using the PyMol software (version 1.5.0.3). The full-length genes of the influenza virus NP protein from 35 different types of the virus were obtained from the GISAID database. Using the MEGA 7.0 software, the sequences of the NP proteins of the selected virus strains were compared to see how much the antigenic epitopes were conserved.

### 2.13. Statistical Analysis

This study used the GraphPad Prism software (version 9.0.2.161) to conduct the statistical analysis. T-tests were employed to analyze statistical significance, with * *p* < 0.05 indicating a significant difference, ** *p* < 0.01 indicating a highly significant difference, and *** *p* < 0.001 indicating an extremely significant difference.

## 3. Results

### 3.1. Virus Isolation and Identification

**Figure 1:**
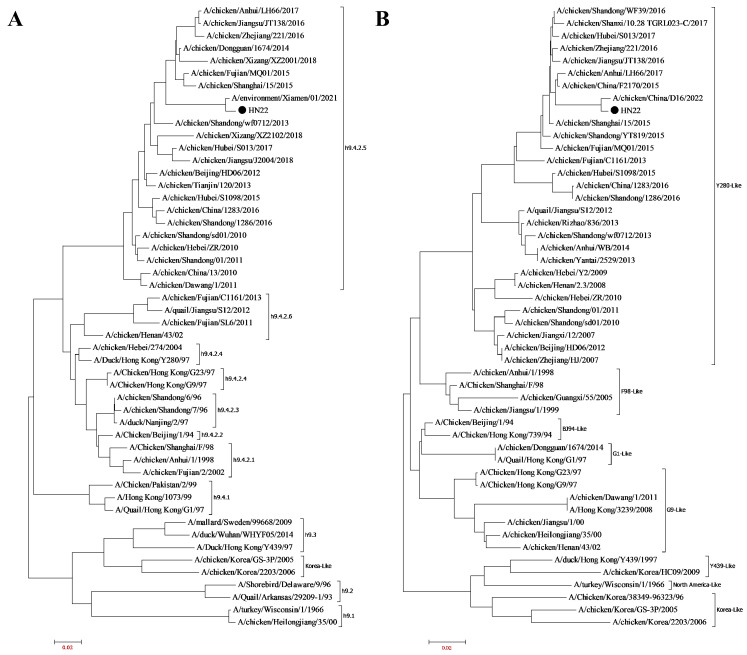
Phylogenetic evolutionary tree of the H9N2-HN22 strain. (**A**) The phylogenetic evolutionary tree of the HA gene. (**B**) The phylogenetic evolutionary tree of the NA gene. Information on the HA genes of representative H9 subtype viruses and the NA genes of representative N2 subtype viruses was downloaded from the National Center for Biotechnology Information database. MEGA 7.0 was used to analyze the gene sequences to obtain the evolutionary tree. ●: the isolated strain H9N2-HN22.

The diseased material was collected and inoculated via allantoic cyst. The allantoic fluid HA titer was 9 log2. The results of the whole-genome sequencing showed that this virus was an H9N2 subtype of avian influenza virus. This strain was named H9N2-HN22 strain (A/chicken/China/HN22/2022). The Reed–Muench method showed that the EID_50_ value was 10^−8.0^/0.1 mL, the ELD_50_ value was 10^−8.0^/0.1 mL, and the TCID_50_ value was 10^−5.1^/0.1 mL. A phylogenetic evolutionary tree for the HA gene and the NA gene was made by using the MEGA 7.0 software. Phylogenetic analysis indicated that the HA gene belonged to the h9.4.2.5 lineage, and the NA gene belonged to the Y280 lineage (Figure 1).

### 3.2. Generation of mAbs Against H9N2-HN22 Strain

To make mAbs against H9N2 AIVs, 6-week-old BALB/c mice were subcutaneously immunized with the inactivated H9N2-HN22 strain. Then, the SP2/0 myeloma cells were mixed with splenocytes from immunized mice using a substance called PEG 1500. Antibody-positive hybridoma cell lines were screened using an ELISA with inactivated viruses as coating antigens. By using a process called limited dilution subcloning, one stable and continuously antibody-secreting cell lines were identified, namely, 2D8. The OD_450_ value of the hybridoma supernatant measured by the established ELISA method was 2.634, and the titer was 1:80,000.

### 3.3. Identification of 2D8 mAb Characteristics

**Figure 2:**
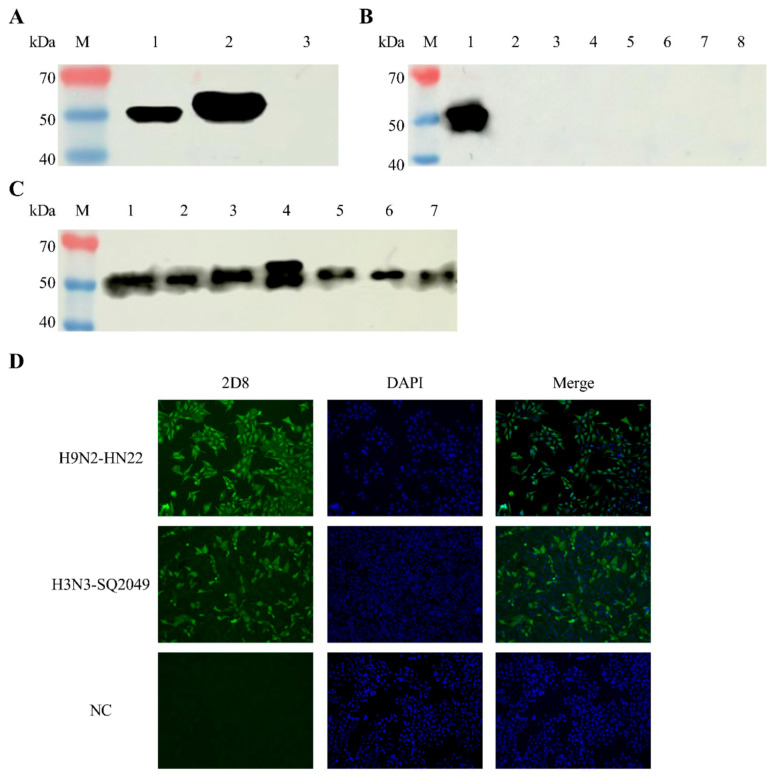
Specificity of the 2D8 mAb as determined by Western blot and IFA. (**A**) Determination of the reactivity of 2D8 mAb with NP proteins; lane M, protein marker; lane 1, H9N2-HN22 strain allantoic fluid; lane 2, H9N2-HN22 NP protein; lane 3, H9N2-HN22 HA1 protein. (**B**) Determination of the reactivity of 2D8 mAb with different viruses; lane M, protein marker; lane 1, H9N2-HN22 strain allantoic fluid; lane 2, FAdV-WZ strain allantoic fluid; lane 3, DAdV-GD2588 strain allantoic fluid; lane 4, FAdV-HN1472 strain allantoic fluid; lane 5, EDSV-76 strain allantoic fluid; lane 6, IBV-C2023-03 strain allantoic fluid; lane 7, CIAV-1412 strain allantoic fluid; lane 8, NDV-La Sata strain allantoic fluid. (**C**) Determination of the reactivity of 2D8 mAb with different subtypes of influenza virus; lane M, protein marker; lane 1, H1N1 subtype strain (H1N1-ZC90) allantoic fluid; lane 2, H3N3 subtype strain (H3N3-SQ2049) allantoic fluid; lane 3, H5 Re-13 subtype strain allantoic fluid; lane 4, H5 Re-14 subtype strain allantoic fluid; lane 5, H7 Re-4 subtype strain allantoic fluid; lane 6, H9N2 subtype strain (H9N2-HN22) allantoic fluid; lane 7, H9N2 subtype strain (H9N2-SQ2023) allantoic fluid. (**D**) IFA assay for the reactivity of 2D8 mAb with influenza-infected MDCK cells. MDCK cells infected with the H9N2-HN22 strain and the H3N3-SQ2023 strain were incubated with the 2D8 mAb as the primary antibody and Dy Light 488-conjugated goat Anti-mouse IgG (H + L) as the secondary antibody (green). Uninfected MDCK cells were used as a negative control. Nuclei were stained with DAPI (blue). Western blot images were acquired with a Western blot imaging system. Fluorescence images were acquired with a fluorescence microscope.

To understand the characteristics of 2D8 mAb, a series of tests was performed, including Western blot, IFA, subtype identification, and ascites titer determination. The results from the Western blot showed that the 2D8 mAb reacted specifically with the H9N2-HN22 NP protein, demonstrating strong specificity. Importantly, it only reacted with the influenza strain and did not cross-react with other viral pathogens. Moreover, it displayed that it can fight off many different types of flu (Figure 2A–C and Figures S1–S3).

**Figure 3:**
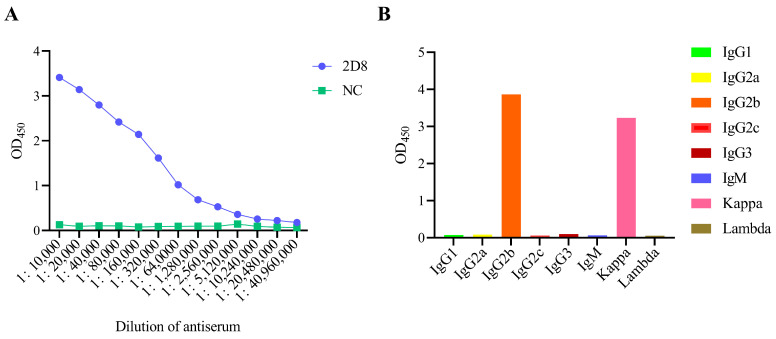
2D8 mAb mouse ascites titer and subtype identification. (**A**) ELISA detection of 2D8 mAb mouse ascites titers. Using ELISA plates coated with inactivated H9N2-HN22 virus, the 2D8 mAb mouse ascites was diluted from a 1:10,000 ratio with 5% skimmed milk for the ELISA titer determination. The result was based on measurement of the OD at 450 nm. (**B**) The 2D8 mAb subtypes were identified using the mouse mAb isotyping ELISA kit. The ELISA results were based on measurement of the OD at 405 nm.

The IFA results confirmed that the 2D8 mAb reacted strongly with cells infected with H9N2-HN22 and H3N3-SQ2049 (Figure 2D and Figure S4). The ascites titer of 2D8 mAb detected by indirect ELISA reached an impressive level of 1:20,480,000 (Figure 3A). Also, 2D8 mAb’s heavy chain is IgG2b, and its light chain type is a kappa chain (Figure 3B).

### 3.4. Peptide Truncation Strategies

**Figure 4:**
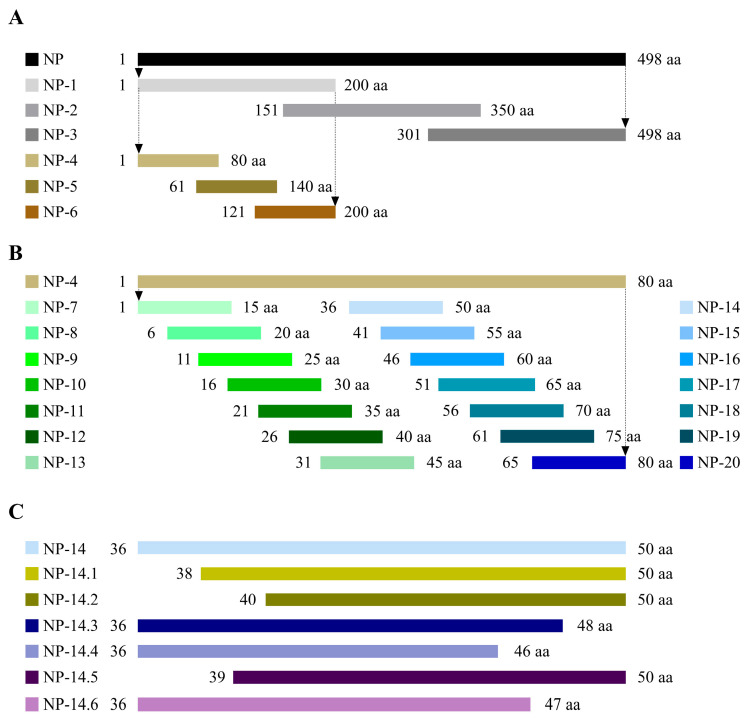
Construction strategy of H9N2-HN22 NP protein truncated peptides. (**A**) First, the H9N2-HN22 NP protein was truncated into three segments, each approximately 200 aa, and they were, respectively, named NP-1, NP-2, and NP-3. These fragments were copied into the pET-28a (+) vector and then transformed into BL21 (DE3) for expression. Then, NP-1 was truncated into three segments, each approximately 80 aa, and they were, respectively, named NP-4, NP-5, and NP-6, cloned into the pET-32a (+) for expression. (**B**) NP-4 was shortened into 14 segments of 15-amino-acid peptides, and they were named NP-7 to NP-20, respectively. (**C**) Different numbers of aa at the C-terminal and N-terminal of NP-14 were shortened, respectively, to precisely locate the epitope targeted by 2D8 mAb. arrows: this sequence is truncated again; aa: amino acid.

To find out which parts of the H9N2-HN22 NP protein are recognized by the mAb, the NP protein was cut into different lengths and used as the antigen (Figure 4). At first, the H9N2-HN22 NP protein was split into three parts, each with 200 amino acids (aa), and expressed in the prokaryotic expression system. According to the results of the Western blot, it was further divided into three segments of 80 aa each (Figure 4A). After the second truncation by Western blot, the NP protein was further truncated into 14 overlapping peptides, with each peptide made up of 15 aa (Figure 4B). Based on the results of the third truncation, we were able to accurately identify the region targeted by the mAb by truncating both ends of the NP protein (Figure 4C).

### 3.5. Identification of Antigenic Epitopes in 2D8 mAb

**Figure 5:**
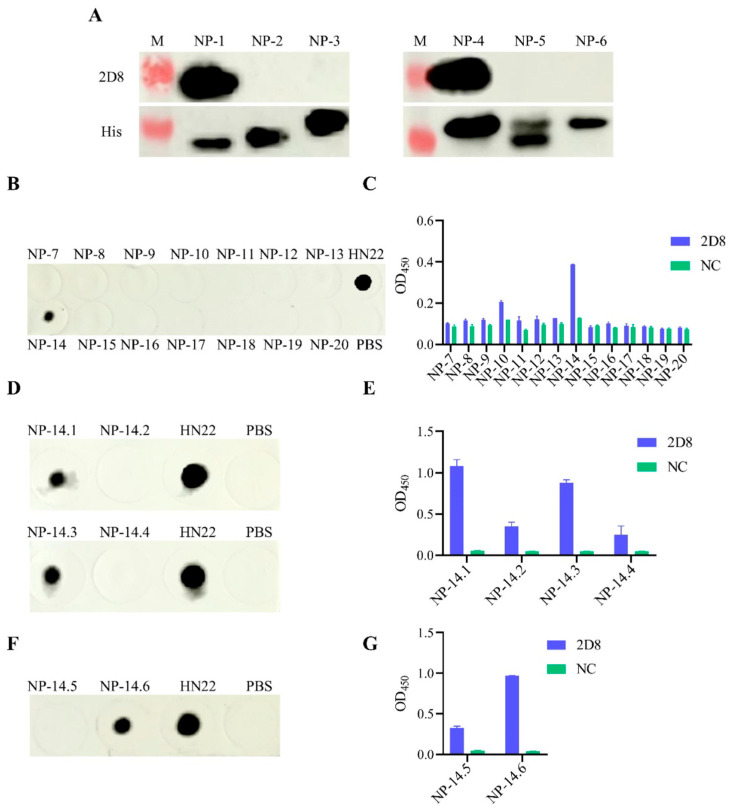
Identification of antigenic epitopes in 2D8 mAb. (**A**) Binding of the first and second protein truncations to the 2D8 mAb, as determined by Western blot. (**B**,**C**) The binding situation of 2D8 mAb to the truncated peptide segments from NP-7 to NP-20. (**D**,**E**) The binding situation of 2D8 mAb to the truncated peptide segments from NP-14.1 to NP-14.4. (**F**,**G**) The binding situation of 2D8 mAb to the last truncated NP-14.5 and NP-14.6 peptide segments. (**B**,**D**,**F**) NP truncated peptides and 2D8 mAb dot blot reaction. (**C**,**E**,**G**) ELISA reaction of NP truncated peptides and 2D8 mAb. The positive control for the dot blot assay was prepared using the allantoic fluid of the H9N2-HN22 strain, while the negative control was prepared using PBS. The ELISA negative control was prepared using the ascites of negative BALB/c mice. Western blot images were acquired with a Western blot imaging system. Dot blot images were acquired with a Western blot imaging system. The ELISA results were based on measurement of the OD at 450 nm.

The shortened proteins made from H9N2-HN22 NP protein, called NP-1, NP-2, and NP-3, were prepared, respectively, and identified by using a special antibody, 2D8 mAb. The results showed that only part of NP-1 specifically bound to 2D8 mAb, suggesting that the antigenic epitopes are between amino acids 1 and 200 (Figure 5A and Figures S5–S8). This region was later split into three parts called NP-4, NP-5, and NP-6. The results showed that the 2D8 mAb only specifically interacted with the NP-4 fragment, suggesting that the antigenic epitope of the 2D8 mAb was located at 1–80 aa (Figure 5A). Then, fragment NP-4 was split into 14 overlapping peptide segments to see how well it could bind to the mAb. The results demonstrated that one mAb specifically attached to a part of the NP-14 (Figure 5B,C and Figure S9). After this, we cut the peptide segment NP-14 at both the N-terminal and C-terminal ends by either two or four amino acids, respectively. The results showed that the mAb only reacted with the NP-14.1 peptide and the NP-14.3 peptide. These were truncated by 2 aa at the N-terminal and 2 aa at the C-terminal, respectively (Figure 5D,E, Figures S10 and S11). Finally, 1 aa was truncated from the N-terminus of the NP-14.1 peptide and from the C-terminus of the NP-14.3 peptide. The results showed that this monoclonal antibody only reacted with the 1-aa truncated C-terminal region of the NP-14.3 peptide, namely, the NP-14.6 peptide (Figure 5F,G and Figure S12). Finally, it was concluded that the epitope targeted by the 2D8 mAb is ^38^RFYIQMCTEL^47^.

### 3.6. Spatial Structure of Epitope

**Figure 6:**
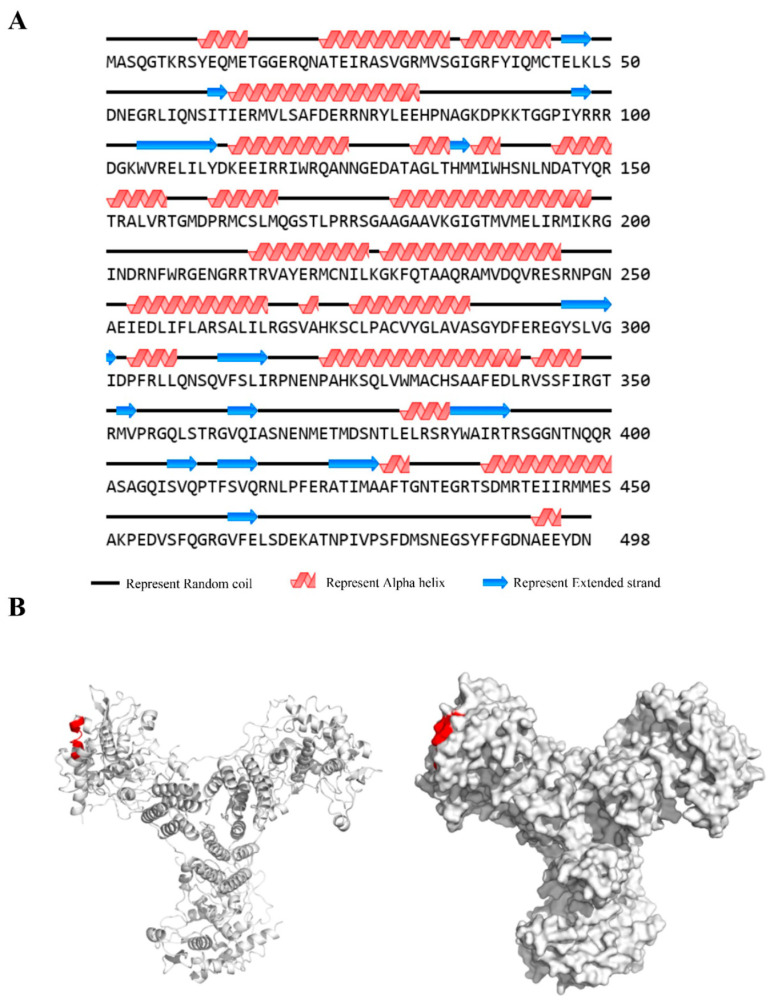
Spatial structures of epitopes in H9N2-HN22 NP protein. (**A**) Predicted secondary structures of the antigenic epitope. Online websites (https://novopro.cn/tools/secondary-structure-prediction.html (accessed on 18 May 2025)) were used to predict the secondary structure of the antigenic epitope. (**B**) Simulated three-dimensional structure of the predicted NP protein shows the epitope ^38^RFYIQMCTEL^47^ (red portion) exposed on the surface of the NP protein. (**Left**): Ribbon representation of the NP protein structure. (**Right**): Structural model of the NP protein trimer. The crystal structure information was downloaded from the Protein Data Bank (PDB ID: 2Q06).

The website https://novopro.cn/tools/secondary-structure-prediction.html (accessed on 18 May 2025) was used to predict the secondary structure of the NP protein. The results revealed that the epitope ^38^RFYIQMCTEL^47^ formed an α-helix, a random coil structure, and a structurally stable β-folding (Figure 6A). The linear B-cell epitope that was found was shown on the 3D structure of the NP protein that had been made using the PyMol software (version 1.5.0.3). It was found that a part of the NP protein structure was exposed on the surface of the NP protein (Figure 6B). This part of the NP protein is called the antigenic epitope ^38^RFYIQMCTEL^47^ (red portion).

### 3.7. Conservative Analysis of Identified Epitope

**Figure 7:**
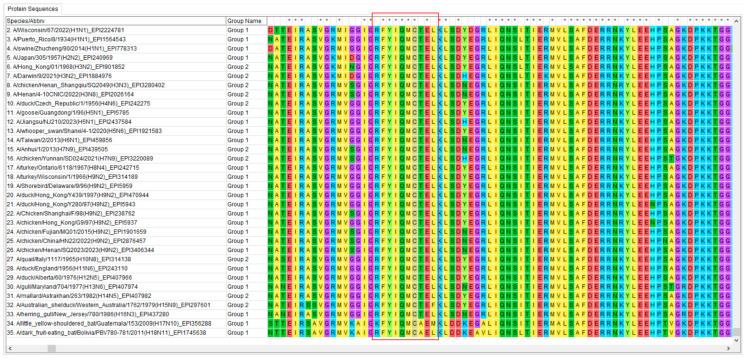
Conservative analysis of identified epitope. The NP protein sequences of 35 international influenza A viruses were compared. A total of 35 influenza A virus strains, covering all 18 HA subtypes, were retrieved from the GISAID database. These strains were isolated from various countries and regions. The part in the red box represents the epitope ^38^RFYIQMCTEL^47^ targeted by the 2D8 mAb that was selected. *: completely conserved site; colors: “chemical properties/types” classification of amino acids, amino acids of the same color represent those with similar physical and chemical properties; letters: the single-letter abbreviations of 20 natural amino acids.

A comparison of the NP protein sequences of 35 different types of the influenza A virus, from both inside and outside of China, using the MEGA 7.0 software, showed that the NP-^38^RFYIQMCTEL^47^ sequence was highly conserved among these 35 strains. It is interesting to note that only three strains showed changes to the amino acids. This conserved clade encompasses the overwhelming majority of subtypes within Group 1 and Group 2. These influenza viral strains, encompassing H1 to H16, exhibit stability, irrespective of their host origins and isolation time points (Figure 7).

## 4. Discussion

Avian influenza viruses (AIVs) are characterized by high variability, rapid transmission, and elevated mortality rates, seriously threatening the development of the poultry industry and human life safety. The surface proteins of AIV, that is, HA, NA, and M2, have long been the primary targets for the development of antiviral strategies [35,36]. Nevertheless, recent investigations have demonstrated that novel vaccines targeting viral internal proteins exhibit more favorable antiviral efficacy [37,38]. NP is the core component of the vRNP complex, indispensable for the entire AIV life cycle [23]. Accumulating evidence indicates that anti-NP monoclonal antibodies mediate antiviral activity by inhibiting vRNP nuclear import as well as viral transcription and replication within the nucleus [39,40]. The high conservation and abundant expression of NP during viral infection render it an optimal antigenic target for the diagnosis of multiple influenza virus subtypes [41,42]. In the present study, we screened a highly broad-spectrum anti-NP monoclonal antibody, 2D8, whose recognized epitope is highly conserved across most AIV subtypes.

In this study, we successfully identified a stable monoclonal antibody (2D8 mAb) targeting the NP protein of the H9N2 subtype AIV through the immunopurification of the HN22 strain. The reactivity of this 2D8 mAb with the H9N2-HN22 strain was confirmed using various techniques, including ELISA, Western blot, and IFA. The results revealed that 2D8 mAb specifically recognizes the H9N2-HN22 strain, and, importantly, it demonstrated strong reactivity against several other influenza A subtypes, including H1, H3, H5, H7, and H9, without cross-reacting with other common poultry viruses. This broad-spectrum reactivity is consistent with the known conservation of NP across different influenza subtypes, making this antibody a valuable tool for diagnostic and surveillance applications. This cross-reactivity is especially significant as it contrasts with other NP-targeting monoclonal antibodies (e.g., 3A4 and 3G2) that are limited to human influenza viruses or specific subtypes [27,43].

Further analysis identified the specific antigenic site of 2D8 mAb as the peptide ^38^RFYIQMCTEL^47^ within the NP protein. Conservation analysis of this epitope across 35 representative influenza A strains showed remarkable stability, surpassing that of many HA and NA epitopes. This high level of conservation makes it a promising candidate for inclusion in universal influenza vaccines, particularly those aiming to provide cross-protection against diverse influenza A subtypes.

NP is a critical target for cytotoxic T lymphocytes (CTLs), which play a crucial role in inducing cellular immunity and cross-protective responses across different viral lineages [25,44,45]. Several studies have demonstrated that NP-based vaccines, such as those using adenovirus vectors or the AIP-C5 vaccine, can offer protection against influenza viruses by stimulating robust T-cell responses [30]. The OVX836 vaccine, which uses NP, has been shown to induce NP-specific lung CD8+ tissue-resident memory (TRM) cells, providing long-term protection against influenza [46]. The epitope identified in this study (^38^RFYIQMCTEL^47^) can be presented by MHC-I molecules on antigen-presenting cells (APCs), triggering a T-cell receptor (TCR)-mediated response and subsequently activating CD8+ T lymphocytes [47,48,49,50,51]. While immunization with peptides containing this sequence has shown partial protection (25%), it highlights the potential for developing a minimal epitope-based vaccine, especially if combined with multiple epitopes or fused with carrier proteins and potent adjuvants to enhance immune responses. This epitope, although a B-cell epitope, also acts as a T-cell epitope, indicating its potential to induce both humoral and cellular immunity.

Nevertheless, this study still has certain limitations. The broad-spectrum reactivity validation of the 2D8 monoclonal antibody (mAb) only encompassed a subset of host-derived strains of major influenza A virus subtypes (H1, H3, H5, H7, and H9), with insufficient coverage of influenza A viruses from diverse hosts (e.g., humans, wild birds, and non-human primates) and emerging recombinant strains. This deficiency may restrict the translational applicability of the 2D8 mAb in clinical diagnosis and therapeutic interventions. However, our current findings have confirmed that the 2D8 monoclonal antibody possesses notable broad-spectrum activity, and the epitope it recognizes is highly conserved among most AIV subtypes. Therefore, based on the current research progress, subsequent experiments will focus on establishing 2D8-based diagnostic assays to systematically evaluate its diagnostic performance against commercially available diagnostic platforms.

## 5. Conclusions

This study successfully screened a monoclonal antibody (2D8 mAb) targeting the NP protein of the H9N2 subtype AIV and identified its conserved B-cell epitope. The mAb exhibits broad-spectrum reactivity with multiple influenza A subtypes, underscoring its potential as a universal diagnostic tool for influenza A virus detection. More importantly, the identified epitope has been shown to be both a B-cell and a T-cell epitope, suggesting its potential for inclusion in universal influenza vaccines. These findings represent a significant step toward overcoming the limitations of current strain-specific vaccines and developing broadly protective, next-generation influenza vaccines, which could play a pivotal role in pandemic preparedness and global influenza surveillance.

## Figures and Tables

**Table 1:** Primers of the truncated H9N2-HN22 NP gene for PCR cloning.

Gene	Primers	Primer Sequences (5′–3′)	Region	Gene Length
NP	NP-F	CGCGGATCCATGGCGTCTCAAGGCACCAAACGAT	1–498 aa	1–1494 bp
	NP-R	CCCAAGCTTTTAATTGTCATATTCCTCTGCATTG		
NP-1	NP-1-F	CGCGGATCCATGGCGTCTCAAGGCACCAAACGAT	1–200 aa	1–600 bp
	NP-1-R	CCCAAGCTTTTAACCTCGTTTTATCATTCGAATC		
NP-2	NP-2-F	CGCGGATCCACGAGAGCTCTTGTACGTACTGGAA	151–350 aa	451–1050 bp
	NP-2-R	CCCAAGCTTTTATGTCCCTCTGATGAAACTTGAG		
NP-3	NP-3-F	CGCGGATCCATAGACCCTTTCCGTCTGCTTCAAA	301–498 aa	901–1494 bp
	NP-3-R	CCCAAGCTTTTAATTGTCATATTCCTCTGCATTG		
NP-4	NP-4-F	CGCGGATCCATGGCGTCTCAAGGCACCAAACGAT	1–80 aa	1–240 bp
	NP-4-R	CCCAAGCTTTTATTCCAGATATCTGTTCCTTCTT		
NP-5	NP-5-F	CGCGGATCCATAACAATAGAGAGAATGGTACTCT	61–140 aa	181–420 bp
	NP-5-R	CCCAAGCTTTTAGTGCCATATCATCATATGGGTA		
NP-6	NP-6-F	CGCGGATCCCGTCAAGCGAACAATGGAGAAGACG	121–200 aa	361–600 bp
	NP-6-R	CCCAAGCTTTTAACCTCGTTTTATCATTCGAATC		

Note: Underlines are restriction endonuclease sites, detailed as follows: GGATCC, *Bam*H I; *AAGCTT*, *Hin*d III. aa: amino acid; bp: base pair.

## Data Availability

The original contributions presented in this study are included in this article and the Supplementary Material. Further inquiries can be directed to the corresponding authors.

## Supplementary Materials

The following supporting information can be downloaded at https://www.mdpi.com/article/10.3390/vetsci13010045/s1. Figure S1: Determination of the reactivity of 2D8 mAb with NP proteins (Lane 1: H9N2-HN22 strain allantoic fluid (~56 kDa); Lane 2: H9N2-HN22 NP protein (~60 kDa); Lane 3: H9N2-HN22 HA1 protein (~53 kDa), Figure S2: Determination of the reactivity of 2D8 mAb with different viruses (Lane 1: H9N2-HN22 strain allantoic fluid (NP protein:~56kDa); Lane 2: FAdV-WZ strain allantoic fluid (~56kDa); Lane 3: DAdV-GD2588 strain allantoic fluid (~56kDa); Lane 4: FAdV-HN1472 strain allantoic fluid (~56kDa); Lane 5: EDSV-76 strain allantoic fluid (~56kDa); Lane 6: IBV-C2023-03 strain allantoic fluid (~56kDa); Lane 7: CIAV-1412 strain allantoic fluid (~56kDa); Lane 8: NDV-La Sata strain allantoic fluid (~56kDa)), Figure S3: Determination of the reactivity of 2D8 mAb with different subtypes of influenza virus (Lane 1: H1N1 subtype strain (H1N1-ZC90) allantoic fluid; Lane 2: H3N3 subtype strain (H3N3-SQ2049) allantoic fluid; Lane 3: H5 Re-13 subtype strain allantoic fluid; Lane 4: H5 Re-14 subtype strain allantoic fluid; Lane 5: H7 Re-4 subtype strain allantoic fluid; Lane 6: H9N2 subtype strain (H9N2-HN22) allantoic fluid; Lane 7: H9N2 subtype strain (H9N2-SQ2023) allantoic fluid).(NP protein: ~56kDa), Figure S4: IFA assay for the reactivity of 2D8 mAb with influenza infected MDCK cells, Figure S5: After the first truncation, the expression was identified using 2D8 primary antibody (Lane 1: NP-1 (~26 kDa); Lane 2: NP-2 (~26 kDa); Lane 3: NP-3 (~26 kDa)), Figure S6: After the first truncation, the expression was identified using His primary antibody (Lane 1: NP-1 (~26 kDa); Lane 2: NP-2 (~26 kDa); Lane 3: NP-3 (~26 kDa)), Figure S7: After the second truncation, the expression was identified using 2D8 primary antibody (Lane 1: NP-4 (~27 kDa); Lane 2: NP-5 (~27 kDa); Lane 3: NP-6 (~26 kDa)), Figure S8: After the second truncation, the expression was identified using His primary antibody (Lane 1: NP-4 (~27 kDa); Lane 2: NP-5 (~27 kDa); Lane 3: NP-6 (~26 kDa)), Figure S9: The binding situation of 2D8 mAb to the truncated peptide segments from NP-7 to NP-20. (1: NP-7; 2: NP-8; 3: NP-9; 4: NP-10; 5: NP-11; 6: NP-12; 7: NP-13; 8: HN22; 9: NP-14; 10: NP-15; 11: NP-16; 12: NP-17; 13: NP-18; 14: NP-19; 15: NP-20; 16: PBS), Figure S10: The binding situation of 2D8 mAb to the truncated peptide segments from NP-14.1 and NP-14.2. (1: NP-14.1; 2: NP-14.2; 3: HN22; 4: PBS), Figure S11: The binding situation of 2D8 mAb to the truncated peptide segments from NP-14.3 and NP-14.4. (1: NP-14.3; 2: NP-14.4; 3: HN22; 4: PBS), Figure S12: The binding situation of 2D8 mAb to the last truncated NP-14.5 and NP-14.6 peptide segments. (1: NP-14.5; 2: NP-14.6; 3: HN22; 4: PBS).
