# Supplementary material for: Highly Conserved Influenza A Nucleoprotein as a Target for Broad-Spectrum Intervention: Characterization of a Monoclonal Antibody with Pan-Influenza Reactivity
Source: Vet Sci. 2026 Jan 3;13(1):45. doi: 10.3390/vetsci13010045 (PMC12846640; doi:10.3390/vetsci13010045)
Supplement: Supplementary file 1 [file vetsci-13-00045-s001.zip › vetsci-4054622-supplementary.pdf]

Supplementary Materials

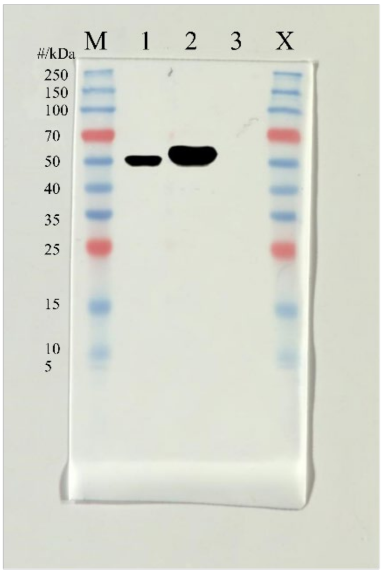

Figure S1 Determination of the reactivity of 2D8 mAb with NP proteins (Lane 1: H9N2-HN22 strain allantoic fluid (~56 kDa); Lane 2: H9N2-HN22 NP protein (~60 kDa); Lane 3: H9N2-HN22 HA1 protein (~53 kDa).

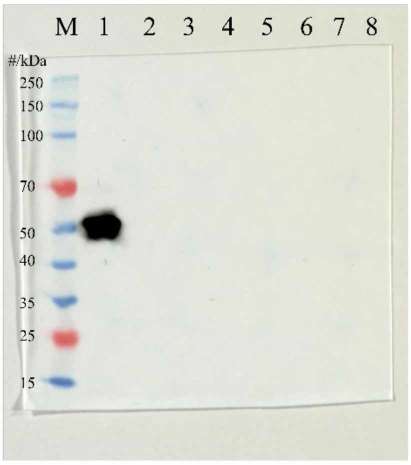

Figure S2 Determination of the reactivity of 2D8 mAb with different viruses (Lane 1: H9N2-HN22 strain allantoic fluid (NP protein:~56kDa); Lane 2: FAdV-WZ strain allantoic fluid (~ 56kDa); Lane 3: DAdV-GD2588 strain allantoic fluid (~ 56kDa); Lane 4: FAdV-HN1472 strain allantoic fluid (~ 56kDa); Lane 5: EDSV-76 strain allantoic fluid (~ 56kDa); Lane 6: IBV-C2023-03 strain allantoic fluid (~ 56kDa); Lane 7: CIAV-1412 strain allantoic fluid (~ 56kDa); Lane 8: NDV-La Sata strain allantoic fluid (~ 56kDa)).

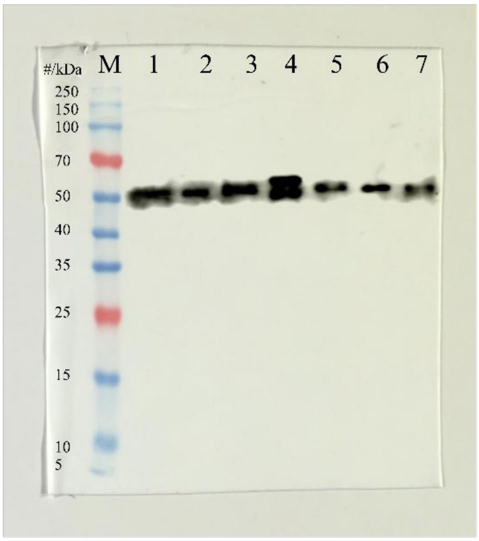

Figure S3 Determination of the reactivity of 2D8 mAb with different subtypes of influenza virus (Lane 1: H1N1 subtype strain (H1N1-ZC90) allantoic fluid; Lane 2: H3N3 subtype strain (H3N3-SQ2049) allantoic fluid; Lane 3: H5 Re-13 subtype strain allantoic fluid; Lane 4: H5 Re-14 subtype strain allantoic fluid; Lane 5: H7 Re-4 subtype strain allantoic fluid; Lane 6: H9N2 subtype strain (H9N2-HN22) allantoic fluid; Lane 7: H9N2 subtype strain (H9N2-SQ2023) allantoic fluid).(NP protein: ~56kDa)

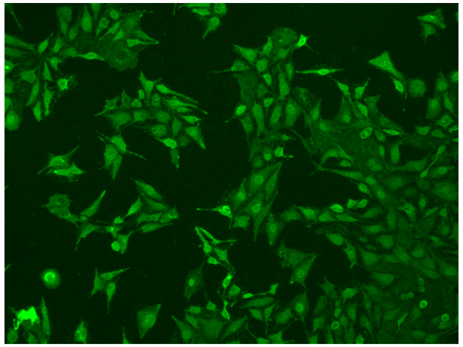

H9N2-HN22 2D8

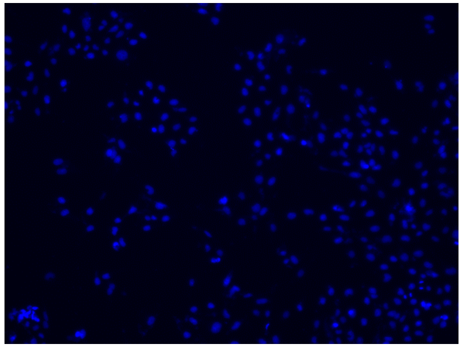

H9N2-HN22 DAPI

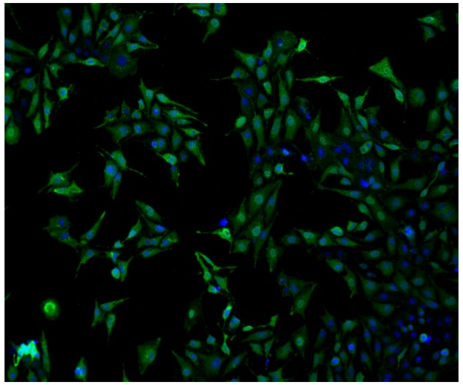

H9N2-HN22 Merge

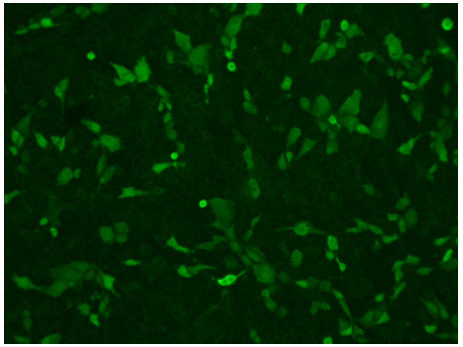

H3N3-SQ2049 DAPI

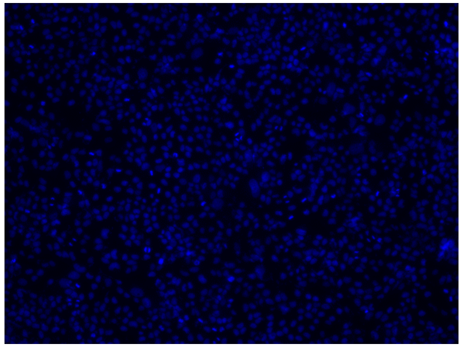

H3N3-SQ2049 2D8

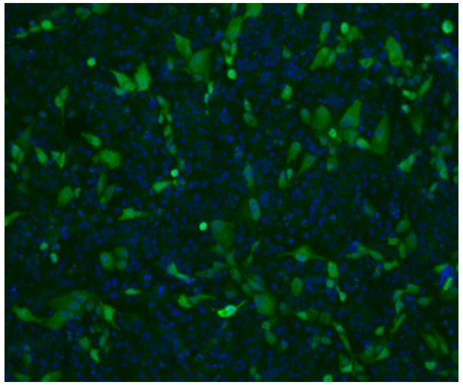

H3N3-SQ2049 Merge

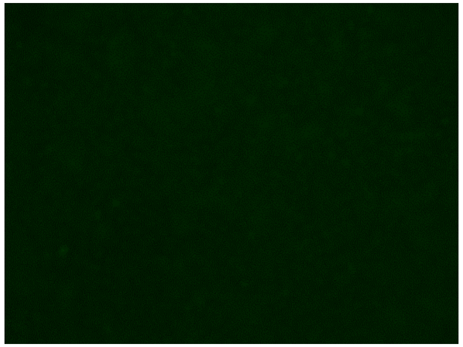

NC 2D8

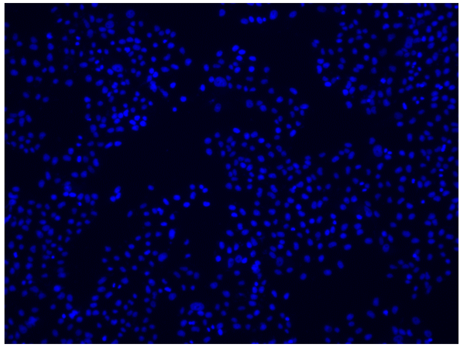

NC DAPI

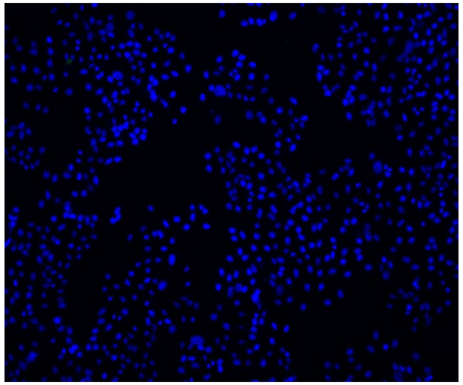

NC Merge

Figure S4 IFA assay for the reactivity of 2D8 mAb with influenza infected MDCK cells.

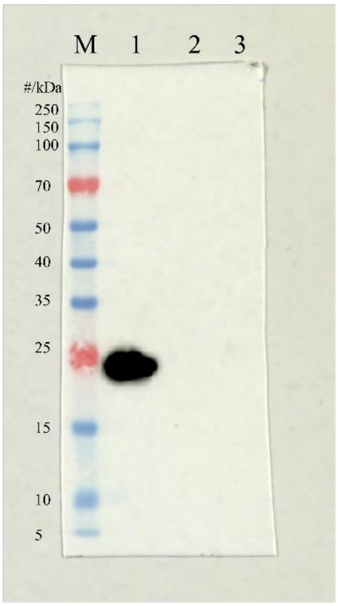

Figure S5 After the first truncation, the expression was identified using 2D8 primary antibody (Lane 1: NP-1 (~ 26 kDa); Lane 2: NP-2 (~ 26 kDa); Lane 3: NP-3 (~ 26 kDa)).

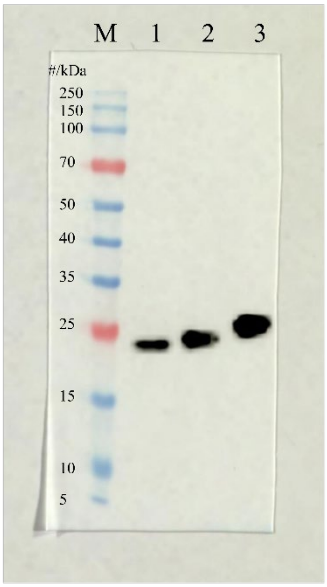

Figure S6 After the first truncation, the expression was identified using His primary antibody (Lane 1: NP-1 (~ 26 kDa); Lane 2: NP-2 (~ 26 kDa); Lane 3: NP-3 (~ 26 kDa)).

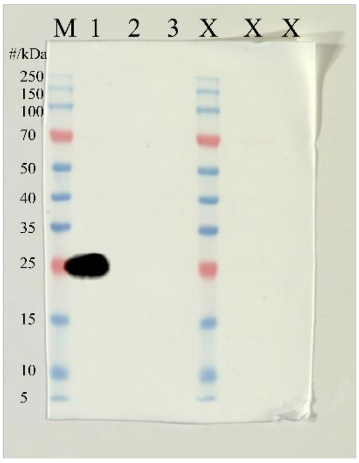

Figure S7 After the second truncation, the expression was identified using 2D8 primary antibody (Lane 1: NP-4 (~27 kDa); Lane 2: NP-5 (~27 kDa); Lane 3: NP-6 (~26 kDa)).

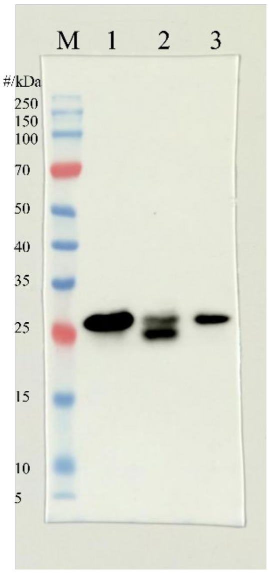

Figure S8 After the second truncation, the expression was identified using His primary antibody (Lane 1: NP-4 (~27 kDa); Lane 2: NP-5 (~27 kDa); Lane 3: NP-6 (~26 kDa)).

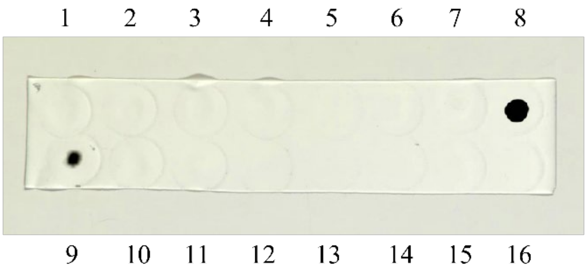

Figure S9 The binding situation of 2D8 mAb to the truncated peptide segments from NP-7 to NP-20. (1: NP-7; 2: NP-8; 3: NP-9; 4: NP-10; 5: NP-11; 6: NP-12; 7: NP-13; 8: HN22; 9: NP-14; 10: NP-15; 11: NP-16; 12: NP-17; 13: NP-18; 14: NP-19; 15: NP-20; 16: PBS)

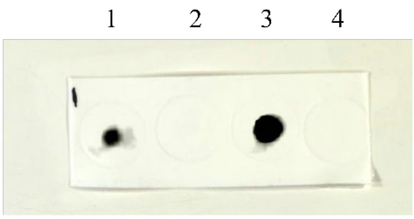

Figure S10 The binding situation of 2D8 mAb to the truncated peptide segments from NP-14.1 and NP-14.2. (1: NP-14.1; 2: NP-14.2; 3: HN22; 4: PBS)

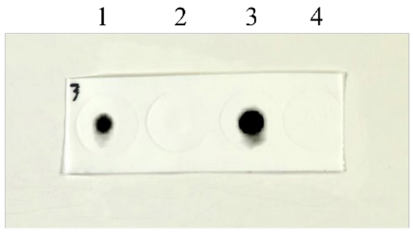

Figure S11 The binding situation of 2D8 mAb to the truncated peptide segments from NP-14.3 and NP-14.4. (1: NP-14.3; 2: NP-14.4; 3: HN22; 4: PBS)

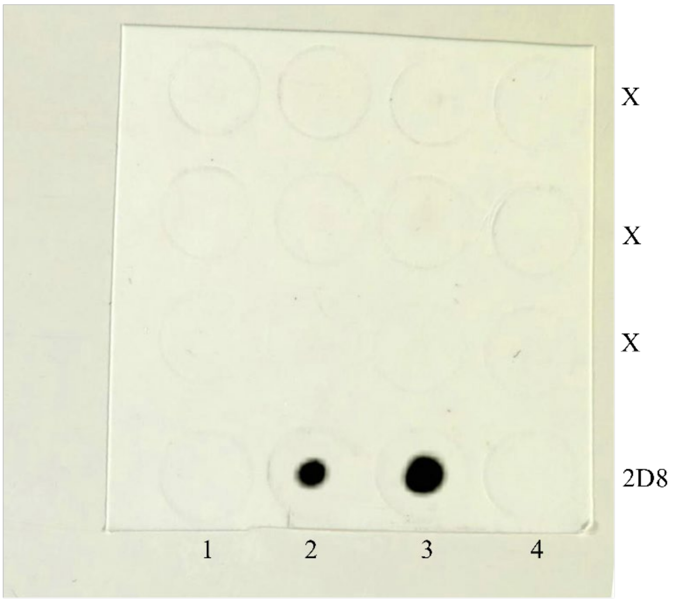

Figure S12 The binding situation of 2D8 mAb to the last truncated NP-14.5 and NP-14.6 peptide segments. (1: NP-14.5; 2: NP-14.6; 3: HN22; 4: PBS)
